# Extracorporeal membrane oxygenation 2016: an update

**DOI:** 10.12688/f1000research.8320.1

**Published:** 2016-04-26

**Authors:** Warwick Butt, Graeme MacLaren

**Affiliations:** 1 Paediatric Intensive Care Unit, Royal Children’s Hospital, Parkville, VIC, 3052, Australia; 2 Department of Paediatrics, University of Melbourne, Melbourne, VIC, Australia; 3 Murdoch Children’s Research Institute, Clinical Sciences, Melbourne, Australia; 4 Cardiothoracic Intensive Care Unit, National University Health System, 5 Lower Kent Ridge Road, 119074, Singapore

**Keywords:** fulminant respiratory failure, neonatal respiratory failure, severe pulmonary hypertension, cardiac failure, mechanical circulatory support, Extra Corporeal Life Support

## Abstract

The use of extracorporeal membrane oxygenation (ECMO) is an important issue for intensivists, critical care nurses, surgeons, cardiologists, and many others. There has been a continued increase in the number of centres performing ECMO. This review examines novel applications and recent trends in the use of ECMO over the last 2 years. These include ECMO to facilitate the safe use of other treatments, changing the timing of initiation, newer equipment and better biocompatibility, and the ability of ECMO programs to essentially choose which cluster of potential complications they are prepared to accept. ECMO continues to evolve, diversify in its applications, and improve in safety.

## Introduction

Extracorporeal membrane oxygenation (ECMO) was developed as a treatment for fulminant respiratory failure (hence its name) in adults in the early 1970s by removing venous blood from the body, adding oxygen, removing carbon dioxide, and returning it to the patient. This therapy was limited to 5 days, and vascular access was obtained by cannulation of the femoral artery and vein. Subsequently, in the early 1980s, this veno-arterial mode was changed to veno-venous, but only a few centres persisted with the technology because bleeding and poor outcomes were common. Similarly, in children, ECMO was first used for neonatal respiratory failure, but, in newborns and young children, severe pulmonary hypertension and poor cardiac function often accompanied respiratory failure. Hence, its use for mechanical circulatory support when isolated cardiac failure occurred was a logical extension. In adults, after cardiac surgery, intra-aortic balloon pumps were commonly used as mechanical circulatory support, but these were very difficult to use successfully in small children (because of the child’s high heart rate and smaller blood vessels). Therefore, veno-arterial ECMO was used in children with cardiac failure, and ECMO became extra-corporeal life support. In the 2000s, a better understanding of the pathophysiology of ECMO and diseases for which it was used led to a rapid re-emergence of this as a therapy for all patients with cardio-respiratory failure; various modifications allowed single or biventricular support, oxygenation or carbon dioxide removal, for short or longer periods of time. This was extensively reviewed in *F1000Prime Reports* 2013; the concluding paragraph in that review stated that “ECMO is a standard therapy in critical care. It is used as a treatment for acute severe cardiorespiratory failure and as a resuscitation strategy in many clinical scenarios. It is also used as a ‘bridge’ to other treatments and transplantation. It continues to be applied to more complex and chronic situations. It is being integrated into multiple-organ support therapies. Substantial improvements in biotechnology and clinical practices over the last 40 years have allowed ECMO to provide a vital role in acute organ support in patients of all ages. It is likely that further such advances will diminish complication rates, facilitate more widespread adoption of the technology in middle- and high-income countries, and improve outcomes from refractory heart, lung, and multiorgan failure” ^1^.

**Figure 1.**
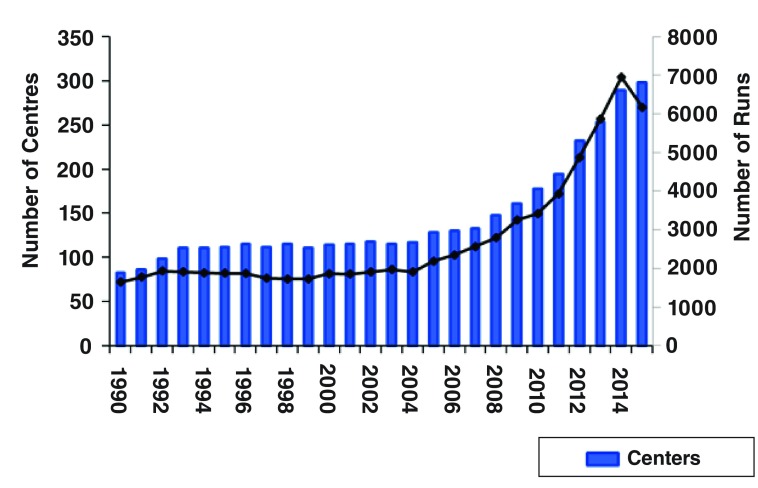
Active extracorporeal membrane oxygenation centres and patients reported to the Extracorporeal Life Support Organization ^20^.

Over the last 2 years, the use of ECMO continues to be an important issue for clinicians: a literature search with ECMO as a key word and including only English language articles and publication dates from 2014 to 2015 yielded 932 articles, 123 reviews, and 16 editorials. During this period, there have also been continued increases in the number of centres performing ECMO ( Figure 1) and in the amount of paediatric and neonatal use in children with cardiac disease as well as a large increase in the use of ECMO for adult respiratory and cardiac disease ( Table 1). The latest cumulative survival reported by the Extracorporeal Life Support Organization is shown in Table 2. The last 2 years have revealed many new uses and issues involving ECMO. These are summarised in the sections below.

**Table 1.** Extracorporeal membrane oxygenation use in runs per year (percent survival) over the last 5 years ^20^.

	2011	2012	2013	2014	2015
Respiratory					
Neonatal and paediatric	1,258 (64%)	1,322 (67%)	1,277 (65%)	1,364 (67%)	1,072 (62%)
Adult	672 (59%)	952 (57%)	1,442 (60%)	1,899 (61%)	1,568 (57%)
Cardiac					
<16 years old	889 (52%)	916 (52%)	978 (50%)	1,063 (50%)	807 (50%)
≥16 years old	604 (37%)	1,033 (41%)	1,253 (42%)	1,679 (43%)	1,769 (41%)

**Table 2.** Cumulative extra-corporeal life support survival ^20^.

	Total patients	Survived extra- corporeal life support	Survived to hospital discharge
**Neonatal**			
Respiratory	28,723	84%	74%
Cardiac	6,269	62%	41%
ECPR	1,254	64%	41%
**Paediatric**			
Respiratory	7,210	66%	58%
Cardiac	8,021	67%	51%
ECPR	2,788	55%	41%
**Adult**			
Respiratory	9,102	66%	58%
Cardiac	7,850	56%	41%
ECPR	2,379	40%	30%

ECPR, extracorporeal cardiopulmonary resuscitation.

## Centres experimenting with new applications of extracorporeal membrane oxygenation

The most important issues in the last few years for clinicians involved in ECMO depend on the type and experience of the ECMO program and the hospital in which ECMO is performed. New programs are focusing on standard uses of ECMO, systems for safe use and deployment of ECMO, management of patients on ECMO, understanding indications and contraindications, education, and simulation. Long-standing programs, on the other hand, are focusing on improving outcomes by considering alternative ECMO strategies (such as normal or high flow for septic shock, different types of peripheral or trans-thoracic cannulation, and initiating ECMO earlier) or different patient groups that hitherto were not considered (such as patients with immune suppression or cancer, pregnant women ^2^, or the elderly ^3^). Perhaps more controversially, some centres have begun research in using it as a bridge to solid organ transplantation ^4^, referred to as extracorporeal support-assisted organ donation. Once a patient has died in a controlled environment as part of a donation-after-cardiac death (DCD) strategy, an aortic balloon is inserted into the proximal descending aorta to prevent re-establishing cerebral blood flow and the patient is cannulated onto femoral-femoral ECMO. This improves abdominal organ metabolic support and has been associated with improved graft survival in the recipient ^5^.

## The use of extracorporeal membrane oxygenation to facilitate the safe use of other treatments

The safety and capacity to transport patients on ECMO now allow the consideration of the use of ECMO as a haemodynamically stable platform in order to facilitate complex surgery ^6^ or interventional cardiology procedures ^7^. Moreover, ECMO stabilises deranged cardiopulmonary physiology in unstable patients such that therapies deemed unsafe—haemodialysis in newborn infants or support of vital organ function during rewarming from accidental hypothermia, such as in avalanche victims ^8– 10^, for example—can be undertaken safely. Comparable to cardiopulmonary bypass facilitating safe cardiac surgery, ECMO provides a haemodynamically stable platform to facilitate these other therapies that otherwise might not be tolerated by the patient. Patients can have complex chemotherapy ^11^ or immunotherapy regimens that cause a severe systemic inflammatory response and are supported with ECMO. ECMO is increasingly being used as a long-term bridge to facilitate lung transplantation ^12, 13^. The use of ECMO in adult cardiorespiratory failure to resuscitate from cardiogenic shock and transport to local cardiac surgical centres for long-term support with ventricular assist devices and transplant programs is also increasingly available ^14, 15^.

## When to initiate extracorporeal membrane oxygenation

The implementation of ECMO is changing as the technology continues to improve. Rather than being used as a last resort or rescue therapy, it has become a standard therapy and increasingly is being implemented earlier in the course of disease in attempts to minimise multi-organ failure. The timing of initiation varies between centres and is a balance of risk for each particular program; it is not a case of better or worse but of the appropriate use of the therapy given each program’s system of use. The realisation that increased survival occurred with earlier use of veno-venous ECMO is now being applied to veno-arterial ECMO in many centres. The role of ECMO in resuscitation and stabilisation of cardiac arrest or cardiogenic shock also varies.

## New equipment for extracorporeal membrane oxygenation: cannulas and biocompatible circuits

Newer cannulas with improved flow characteristics or more biocompatible plastics are being developed that will further improve the safety of ECMO. The ultimate goal is to have a circuit that is fully biocompatible with no need for anticoagulation and no risk of thromboembolism or haemorrhage. Excellent circuit flow characteristics and self-regulated flow-demand loops are only a generation away ^16– 18^.

## Choose your complication

Currently, after weighing the benefits and risks to determine an approach that suits their needs, each program can essentially choose which cluster of potential complications they are prepared to accept. For instance, the type and extent of anticoagulation contribute to the likelihood of promoting either surgical bleeding or thromboembolism. A transthoracic cannulation approach leads to a higher incidence of bleeding and mediastinitis but also allows larger cannulas with increased flow. Jugular-carotid cannulation is more likely to cause cerebral thromboembolic and haemorrhagic complications than central cannulation ^19^. Femoro-femoral cannulation is more likely to have differential hypoxemia or limb ischaemia than other types of cannulation. These are very important issues, as each program has a different cannulation strategy influenced by the skill set of the cannulating doctor; intensivists are different from general surgeons, who are different from cardiac surgeons. Comparison of results clearly requires knowledge of similarities and differences between programs.

## Conclusions

ECMO continues to evolve, diversify in its applications, and improve in safety. Patient outcome is centre specific and very dependent on local factors, local indications, and contraindications. Evaluation of each centre’s results should be done with these factors in mind; the ECMO community continues to share results and knowledge in an attempt to improve patient outcomes.
